# An Overview of Chemical Profiles, Antioxidant and Antimicrobial Activities of Commercial Vegetable Edible Oils Marketed in Japan

**DOI:** 10.3390/foods7020021

**Published:** 2018-02-10

**Authors:** Tran Dang Xuan, Gu Gangqiang, Truong Ngoc Minh, Tran Ngoc Quy, Tran Dang Khanh

**Affiliations:** 1 Graduate school for International Development and Cooperation, Hiroshima University, Hiroshima 739-8529, Japan; ggq353061325@gmail.com (G.G.); minhtn689@gmail.com (T.N.M.); tnquy@ctu.edu.vn (T.N.Q.); 2 Department of Genetic Engineering, Agricultural Genetics Institute, Pham Van Dong Street, Hanoi 122300, Vietnam; khanhkonkuk@gmail.com

**Keywords:** edible oils, antimicrobial activity, antioxidant activity, phenolic acids, flavonoids

## Abstract

This study analyzed chemical components and investigated the antioxidant and antimicrobial activities of fourteen vegetable edible oils marketed in Japan. High-performance liquid chromatography (HPLC) was used to identify and quantify principal phenolic acids and flavonoids. In the 1,1-diphenyl-2-picrylhydrazyl (DPPH) radical scavenging assay, sunflower, safflower, canola, soybean, Inca inchi, sesame, and rice bran showed markedly greater activity, whilst the percentage of lipid peroxidation inhibition (LPI%) in sunflower, canola, cotton, grape, flax, perilla, Inca inchi, perillartine, and rice bran were significantly higher than other oils. Maximum total phenol content (TPC) was recorded in flax, followed by perillartine, rice bran, and perilla, whereas total flavonoid content (TFC) was the greatest in Inca inchi and sesame. Benzoic acid was the most common constituent, followed by vanillic acid, *p*-hydroxybenzoic acid, ferulic acid, and *p*-coumaric acid. On the other hand, luteolin was the most abundant flavonoid, followed by esculetin, myricetin, isoquercetin, and kaempferol, while fisetin was detected only in sunflower. In general, all of the edible oils showed antimicrobial activity, but the growth inhibition of *Staphylococcus aureus* and *Escherichia coli* of cotton, grape, chia, sesame, and rice bran were greater than other oils.

## 1. Introduction 

Edible vegetable oils are important sources of fatty acids (mainly unsaturated), which are predominantly triglycerides (95–98%), whist the remaining (5–2%) consists of complex mixtures of minor compounds such as sterols, triterpenic dialcohols, diglycerides, and squalene [1,2,3,4,5]. Antioxidants and antibacterial activities are key agents for improving oxidative stability. Aluyor and Ori-Jesu [6] noted that the shelf life of edible oils in food uses and their applicability in industry were greatly dependent on their oxidative stability and antibacterial activity. 

Like humans, foods also need to be protected from oxidation. Foods in general have antioxidant additives to avoid lipid oxidation, which results in off-flavor development [7,8,9,10,11,12,13]. As oxidation in foods is a chain process, a trace amount of antioxidant is required to suppress the process of oxidation [14]. Reactive oxygen species (ROS) are formed during normal cellular metabolism, but they become toxic when present in high doses [15]. Caponio et al. [16] reported that polar substances such as triglyceride oligopolymers and oxidized triglycerides found in microwaved were higher in quantity than in conventionally heated olive oil.

In recent years, phenolic acids and flavonoids have been reported to contain multifunctional properties and beneficial effects on human health, among which polyphenols have attracted great attention [17]. Edible oils contain a number of phenolic compounds, which contribute to oxidative stability, and may serve as antioxidants to reduce the stress of oxidation on human health [18]. The DPPH (1,1-diphenyl-2-picrylhydrazyl) radical scavenging activity and *β*-carotene bleaching methods are two of the most commonly methods used to determine antioxidant activities [18]. Phenolic compounds have been known as potential agents for preventing and treating many oxidative stress-related diseases, such as cardiovascular disease, cancer, aging, diabetes mellitus, and neurodegenerative diseases [19,20]. 

Edible oils extracted from plant sources are important in foods and other industries [21]. Oil extracts of sulfur-rich vegetables may have potent antimicrobial activity, and could be used in food preparation to get the synergistic effect of the oils and vegetables [22]. *S. aureus* and *E. coli* are the most common bacteria found in the environment and the human body, and are opportunistic pathogens that cause severe and life-threatening infections in immunocompromised patients [21,22]. Thus, the control of these two bacteria in the food industry is required.

Japan was ranked the eleventh-largest market of vegetable oils in 2016, with US$1.51 billion worth of retail value sales. Between 2012 and 2016, the world vegetable oil products market grew at a compound annual growth rate (CAGR) of 8.16%, while that of Japan was 5.61% [23]. A significant portion (41.3%) of edible oils sold in Japan in 2016 were coconut, grapeseed, groundnut, sesame, and blended oils [23]. It is estimated that by 2021, the retail values of edible oils in Japan may reach US$1.88 billion, and the growth of consumption rate in 2017–2021 is foreseen to be 4.3% CAGR [23]. In this study, the selective criteria of the edible oils were based on their availability in markets. Extra virgin olive oil was not selected because it was not obtainable where samples were collected. 

In this study, the antioxidant activity of the fourteen vegetable edible oils marketed in Japan by the DPPH radical scavenging activity and the *β*-carotene bleaching method were examined. Total contents of phenolics and flavonoids, as well as individual phenolic acids and flavonoids were analyzed and quantified by HPLC. The antimicrobial activity on the growth of *S. aureus* and *E. coli* in laboratorial bioassays were also evaluated. This study aimed to provide information for customers in Japan to have more choices on selection of edible oils available on the markets.

## 2. Materials and Methods

### 2.1. Materials and Standard Chemicals

Fourteen edible oils (sunflower, safflower, canola, soybean, cotton, grape, flax, avocado, chia, Inca inchi, perillartine, sesame, and rice bran oil) were purchased from different supermarkets in Hiroshima Prefecture, Japan, in 2016. Details are described in Table 1. Standard chemicals for the analysis of individual phenolic acids (gallic acid, protocatechuic acid, catechol, chlorogenic acid, *p*-hydroxybenzoic acid, vanillic acid, caffeic acid, syringic acid, vanillin, ferulic acid, sinapic acid, *p*-coumaric acid, benzoic acid, ellagic acid, and cinnamic acid), and flavonoids (esculetin, isoquercetin, myricetin, fisetin, morin, quercetin, luteolin, kaempferol, isohamnetin, apigenin, rhamnetin, and galangin), extracting solvents, and buffers were of analytical grade and purchased from Wako company, Tokyo, Japan.

### 2.2. Extraction of Samples

A volume of 10 mL of each oil was extracted twice with 20 mL of methanol (MeOH) each time. The combined extracts were evaporated to dryness in a rotary evaporator at room temperature. The precipitates were weighed, dissolved in MeOH, and kept in the dark at 4 °C for further analysis. 

### 2.3. Antioxidant Properties 

#### 2.3.1. DPPH Radical Scavenging Activity 

The DPPH scavenging activity was evaluated according to a method described by Elzaawely et al. [24]. An aliquot of 0.5 mL sample extracts was mixed with 0.25 mL of 0.5 mM DPPH and 0.5 mL of 0.1 M acetate buffer (pH 5.5). The mixtures were shaken vigorously and left at room temperature in the dark for 30 min. The reduction of the DPPH radical was determined by reading the absorbance at 517 nm using a spectrophotometer (HACH DR/4000U, Loveland city, CO, USA). MeOH instead of oil extract was used as a control. The percentage of DPPH radical scavenging activity (RSA) was calculated as follows: RSA (%) = 100 × ((abs_control_ − abs_sample_)/abs_control_), where abs_control_ is the absorbance of the reaction without a sample and abs_sample_ is the absorbance of the reaction with samples. The IC_50_ (inhibitory concentration) value was determined as the inhibition of a concentration necessary to decrease the DPPH radical concentration by 50% and expressed in mg/mL. Thus, a lower IC_50_ value indicates higher DPPH radical scavenging activity. All measurements were performed in triplicate. 

#### 2.3.2. Determination of Antioxidant Activity with the *β*-Carotene Bleaching Method

The antioxidant activity was evaluated by the *β*-carotene linoleate bleaching system described by Elzaawely et al. [24]. In detail, a volume of 2 mL of *β*-carotene was dissolved in 10 mL of chloroform and a 1-mL aliquot of a chloroform solution that was mixed with 20 μL of linoleic acid and 200 mg Tween-40. The chloroform solution was evaporated under vacuum at room temperature. Afterwards, an aliquot of 50 mL of oxygenated water was added, and the emulsion was vigorously shaken until complete homogenization was achieved. The obtained emulsion was freshly prepared before each experiment. A MeOH solution of 0.12 mL sample was mixed with 1 mL of the emulsion. An equal amount of MeOH was used as the control. The solutions were incubated at 50 °C and recorded at 492 nm using a spectrophotometer (HACH DR/4000U, Loveland city, CO, USA). All extracts were measured every 30 min up to 180 min. The assays were carried out in triplicate. Lipid peroxidation inhibition (LPI) was calculated using the following equation:

LPI (%) = A_1_/A_0_ × 100

where A_0_ is the absorbance value measured at zero time for the test sample, while A_1_ is the corresponding absorbance value measured after incubation for 180 min. Therefore, a higher LPI value shows a higher antioxidant activity. 

### 2.4. Determination of Total Phenolic Content 

The total phenolic content of the extracts was determined using the Folin–Ciocalteu method described previously [25,26]. Briefly, 0.125 mL of the extracts were mixed with 0.5 mL of distilled water and 0.125 mL of Folin–Cicalteu’s reagent was added. After 6 min, 1.25 mL of 7.5% aqueous Na_2_CO_3_ solution was added. The solution was then adjusted to a final aliquot of 3 mL with distilled water and mixed vigorously. The mixture was incubated for 90 min at room temperature. The absorbance at 760 nm was recorded using a spectrophotometer (HACH DR/4000U-USA). The calibration equation for gallic acid was: *y* = 0.0061*x* + 0.0551 (*r*^2^ = 0.9795), where *y* is the absorbance and *x* is the concentration of gallic acid in mg/mL The total phenolic content was expressed as mg of gallic acid equivalent per gram (GAE/g) oil extract. 

### 2.5. Determination of Total Flavonoid Content (TFC)

The total flavonoid content in the oil extracts was determined using the method described by Djeridane et al. [27]. A volume of 0.5 mL of the sample extract was mixed with 0.5 mL of the 2% aluminum chloride MeOH solution. After 15 min at room temperature, the absorption was measured at 430 nm using a spectrophotometer (HACH DR/4000U-USA), for which MeOH was used as a blank sample. 

### 2.6. Identification of Individual Phenolic Acids and Flavonoids by HPLC

An aliquot of 5 mL of each sample extract was filtered using a 0.2 µm filter (KANTO Chemical, Tokyo, Japan), then injected into an HPLC instrument (JASCO PU-2089 Plus, JASCO Corporation, Tokyo, Japan, column J–Pak Symphonia C18 110A (4.6 mmØ × 15 mm). The analyses of phenolic acids and flavonoids were carried out using HPLC described in Xuan et al. [28] and literature. In details, a solvent system including (A) 0.1% of acetic acid, (B) 100% MeOH, gradient program: 5–10 min, 5–20% (A); 10–30 min, 20–80% (A); 30–40 min, 80–100% (A), wavelength: 254 nm, and flow rate: 1.0 mL/min). Each sample was measured three times, and concentrations of phenolic compounds were identified and quantified in comparison with peak areas of the standards. The individual phenolic acids and flavonoids from extracts were identified and quantified by comparing with retention times and peak areas of the standards, respectively. A calibration curve (linear regression curve) for quantification was established from different dilutions (1, 10, 25, 50, and 100 ppm) of each standard phenolic acids and flavonoids. The equation *Y* = a + b*X* obtained from the calibration curve (0.9 < *r*^2^ < 0) was used to calculate the concentrations of phenolic acids and flavonoids. Of which, *Y* is the peak area of the standard compound, a and b are the known values obtained from the linear regression curve, and *X* is the concentration of the identified phenolic acid/flavonoid need to quantify.

### 2.7. Antimicrobial Activity Test

#### 2.7.1. Agar Preparation

An amount of 8 g LB broth (Lennox) 20 g agar powder was dissolved in 1000 mL distilled water, then sterilized by an autoclave for 20 min at 121 °C. Then, it was cooled to 55 °C and stored at 4 °C in the dark for further experimentation. 

#### 2.7.2. Antimicrobial Test

The antimicrobial activity of the oil extracts was evaluated according to the method described previously [29,30,31]. In this experiment, the LB broth medium as described above was used to grow the bacteria for 24 h at 37 °C. The final populations of the bacteria were standardized to 5.6 × 10^6^ CFU/mL (*S. aureus*) and 1.33 × 10^8^ CFU/mL (*E. coli*). An amount of 0.1 mL of the bacteria suspension was placed on each plate filled with the LB agar. After that, different volumes (0.1, 0.05, and 0.01 mg/mL) of each oil extract were poured on sterile discs (6 mm diameter), covered by filter papers, kept at 37 °C for 24 h, and then the inhibition zone was measured.

### 2.8. Statistical Analysis

The data was analyzed by two-way ANOVA using the Minitab 16.0 software (Sydney, Australia) for Windows. The Tukey’s test was used to indicate significant difference with a level of *p* < 0.05 and expressed as mean ± standard error (SE). The correlation coefficients among the examined factors were also calculated and expressed in linear value (*r*^2^) at *p* < 0.05.

## 3. Results

### 3.1. DPPH Radical Scavenging Activity Assay

The radical scavenging activity of the edible oil extracts was tested using DPPH, which has the advantage of being unaffected by certain side reactions, including metal ion chelation and enzyme inhibition. The antioxidant effect is proportional to the disappearance of the purple color of DPPH in test samples.

As the lower value of IC_50_ indicated stronger DPPH radical scavenging activity, Table 2 showed that the value of antioxidant activity of sunflower was the maximum (0.2 mg/mL); however statistically, the DPPH scavenging activities of safflower, canola, soybean, cotton, grape, Inca inchi, sesame, and rice bran were not significantly different as compared with that of sunflower. Flax and perillartine showed intermediate antioxidant properties, while avocado and chia showed the lowest DPPH radical scavenging activity. 

### 3.2. β-Carotene Bleaching Method

The measurement of the *β*-carotene bleaching method is based on the loss of the yellow color of *β*-carotene due to its reaction with radicals which are formed by linoleic acid oxidation in an emulsion [27]. The antioxidant activity of samples is not only dependent on temperature, but also on many other factors such as the structure, the character of the lipid system, and the binding of the fatty acids [27]. The lipid peroxidation inhibition value (LPI)% was the greatest for grape, followed by cotton, Inca inchi, rice bran, sunflower, canola, perilla, and flax, although their values were not significantly different (Table 2). The lowest value of LPI% was found in chia, followed by avocado. Other oils possessed intermediate values of linoleic acid inhibition. 

### 3.3. Total Phenolic and Flavonoid Contents

The total phenolic content (TPC) and total flavonoid content (TFC) of the edible oils are shown in Table 3. There was a wide variation of the TPC among examined samples, of which flax was the maximum and significantly higher than other oils, followed by perillartine, rice bran, perilla, and grape. Safflower had the lowest value of TPC, whereas that of other edible oils varied between 3.01 and 11.31 mg GAE/g extracts (Table 3). In contrast, the TFC values were not varied as much as the TPC did, of which Inca inchi recorded the maximum, followed by sesame, avocado, flax, and perilla. Besides, perillartine, chia, rice bran, and grape showed TFCs between 0.10 and 0.12 mg rutin equivalent (RE)/g extract, whereas that of the other oils exhibited TFC values <0.07 mg RE/g extracts (Table 3). 

Comparing among the examined edible oils, flax, perillartine, Inca inchi, sesame, rice bran, perilla, grape, and avocado had higher levels of TPC and TFC than those of other oils, although the values did not proportionally parallel between TPC and TFC (Table 3).

### 3.4. Identification and Quantification of Individual Phenolic and Flavonoid Compounds

**Figure 1:**
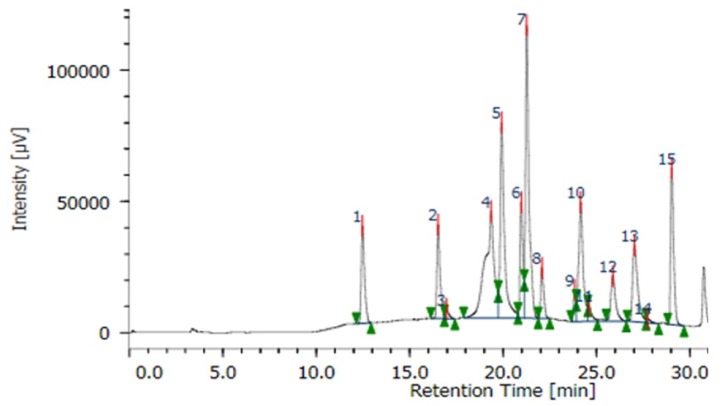
HPLC chromatogram of phenolic acid standards. Peaks 1: gallic acid; 2: protocatechuic acid; 3: catechol; 4: chlorogenic acid; 5: *p*-hydroxybenzoic acid; 6: vanillic acid; 7: caffeic acid; 8: syringic acid; 9: vanillin; 10: ferulic acid; 11: sinapic acid; 12: *p*-coumaric acid; 13: benzoic acid; 14: ellagic acid; 15: cinnamic acid.

Eight phenolic acids were identified, including chlorogenic acid, *p*-hydroxybenzoic acid, vanillic acid, ferulic acid, sinapic acid, *p*-coumaric acid, benzoic acid, and ellagic acid (Figure 1). Benzoic acid and vanillic acid were the most abundant. In quantity, benzoic acid recorded the highest (0.071–0.607 mg/mL extracts), whilst the other phenolic acids were in much lower quantities (0.004–0.079 mg/mL extracts) (Table 4). 

**Figure 2:**
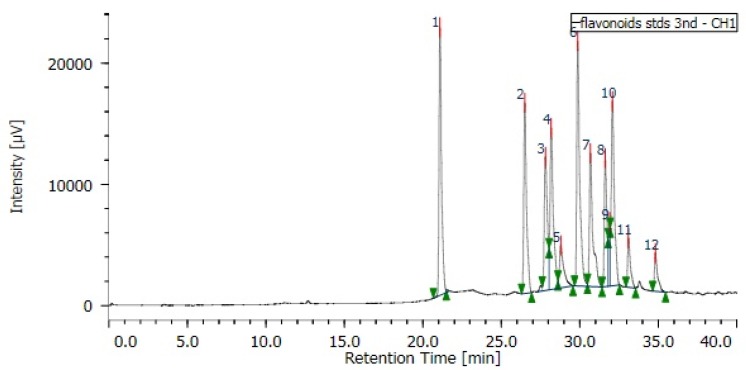
HPLC chromatogram of the flavonoid standards. Peaks 1: esculetin; 2: isoquercetin; 3: myricetin; 4: fisetin; 5: quercetin n-hydrate; 6: quercetin; 7: luteolin; 8: kaempferol; 9: isohamnetin; 10: apigenin; 11: rhamnetin; 12: galangin.

Figure 2 shows the HPLC profile of the 12 flavonoid standards, including esculetin, isoquercetin, myricetin, fisetin, quercetin n-hydrate, quercetin, luteolin, kaempferol, isohamnetin, apigenin, rhamnetin, and galangin. Of them, 7 compounds were identified, consisting of esculetin, isoquercetin, myricetin, fisetin, luteolin, kaempferol, and rhamnetin (Table 5). In details, luteolin was the most abundant. Myricetin and esculetin were found in 7 edible oils, isoquercetin and kaempferol were observed in 5 edible oils, while rhamnetin was detected in 2 edible oils (Table 5). 

These flavonoids were quantified by HPLC (Figure 2), and are shown in Table 5. It was found that the identified flavonoids presented in much greater quantity than that of phenolic acids. In detail, myricetin possessed the maximum (3.986–9.947 mg/mL), followed by rhamnetin (2.127–3.613 mg/mL), whereas kaempferol was the lowest (0.239–0.677 mg/mL). The remaining isoquercetin, esculetin, and luteolin obtained 0.255–3.553 mg/mL (Table 5). Comparing the total amount of flavonoids, perilla showed the highest quantity (12.542 mg/mL), followed by grape and sunflower (10.089 and 10.303 mg/mL, respectively). Safflower had the least (0.28 mg/mL), followed by canola (0.988 mg/mL), whereas both sesame and rice bran had a similar amount (1.53 mg/mL). Other edible oils had 3.192–6.707 mg/mL (Table 5). 

### 3.5. Antimicrobial Activity 

The antimicrobial activity of the examined edible oils is shown in Table 6. It is observed that all oils showed inhibition against growth of the two bacteria, and the inhibitory levels were proportional to the applied doses. In the *S. aureus* trial, at concentrations of 0.01–0.05 mg/mL, although the suppressive magnitudes were all higher than the negative control, no significant difference was observed. At the maximum dose of 0.10 mg/mL, cotton, grape, chia, sesame, and rice bran showed significantly higher levels of inhibition than the negative control. However, sunflower and Inca inchi exerted the least suppression, which was significantly lower than other oils and the positive control. 

In *E. coli*, at the lowest dose of 0.01 mg/mL, no marked difference as compared with the negative control was observed (Table 6). At a higher dose of 0.05 mg/mL, four oils—including soybean, cotton, flax, and sesame—exhibited remarkably higher inhibition than other oils and the negative control. At the greatest concentration of 0.10 mg/mL, although the oils displayed significantly lower antimicrobial activity than the positive control, they were all remarkably greater than the negative control, except sunflower, cotton, and perilla (Table 6). In general, cotton, grape, chia, sesame, and rice bran exhibited the stronger inhibition of the growth of *S. aureus* and *E. coli* than the other oils.

### 3.6. Correlation Coefficients among Analyzed Factors

Results in Table 7 showed the correlation coefficients among total phenolic and flavonoid contents, antioxidant activity (DPPH radical scavenging activity and *β*-carotene bleaching assay), and total amounts of individual phenolic acids and flavonoids. It was observed that the highest correlation coefficient was found between the DPPH radical scavenging and *β*-carotene bleaching activities (r^2^ = 0.401), followed by TPC and IPA (total amount of individual phenolic acids) (r^2^ = 0.3235), and DPPH radical scavenging activity and IPA (r^2^ = 0.3322). The IF (total amount of individual flavonoids) showed a greater correlation coefficient of antimicrobial activity (r^2^ = 0.0709–0.1496) than that of the IPA (r^2^ = 0.0002–0.0003) (Table 7). Total phenolic and flavonoid contents and antioxidant capacity did not show strong correlation coefficient against the antimicrobial activity of the edible oils. In general, no marked differences were observed among analyzed factors (Table 7).

## 4. Discussion

The results showed that the highest amount of total phenolics was in the flax seed oil extract (39.16 mg/g) and was significantly higher than all of other edible oils. Anwar and Przybylski [31] determined a lower content of total phenolics of 27 mg/g in flax seeds. The reason for the different results might be due to a difference in the varieties of flax seeds, extraction solvent, temperature, and equipment [32]. Even if the total phenolic content of flaxseed oil extract was remarkably greater than other edible oils, the IC_50_ of flax seed oil of the DPPH scavenging activity showed a low level at 2.396 mg/mL, and the *β*-carotene bleaching value were significantly higher than the other oils (Table 2), indicating that total phenolics in flax seed oil was not proportional to its antioxidant activity. The antioxidant activity of plant extracts may relate to the presence of some individual phenolic compounds [33]. Inca inchi oil extract showed the maximum amount of total flavonoids (0.34 mg/g); however, the IC_50_ of the DPPH radical scavenging activity of this oil was 0.430 ± 0.007 mg/mL—lower than sunflower, safflower, canola, soybean, and cotton (Table 2). The lowest values of TFC were in safflower and soybean (0.03 mg RE/g oil extract) (Table 3).

Sunflower oil extract gave the best results in the DPPH radical scavenging test, and similar capacity in the *β*-carotene bleaching assay as compared with other oils, except avocado and chia (Table 2). Among dietary plant sources, sunflower seeds are characterized by high antioxidant activity [33]. This oil is shown to have low total phenolic and total flavonoid contents, but the flavonoid compound fisetin was found only in sunflower oil extract. Further, esculetin, isoquercetin, and kaempferol were also identified in sunflower oil extracts and the total amount of the identified individual flavonoids (IF) was higher than other oils, except grape and perilla (Table 5). In addition, *p*-coumaric acid was found in much higher quantity in sunflower than other oils. As a result, fisetin, esculetin, isoquercetin, kaempferol, and *p-*coumaric acid might play an important role in the antioxidant activity of sunflower seed oil. Karamac et al. [33] reported that sunflower seed is an important butyraceous source grown in many countries, particularly in many European countries, to provide rich antioxidants. 

Bail et al. [34] reported that the TPC of grape seed levels was at 70 ± 1.76 μg/g, and Doshi [35] revealed that the TPC of two grape varieties Navarang and Merlot were 65.8 and 41.7 mg/mL, respectively, which was higher than the TPC of grape seed oil examined in this study (15.56 ± 0.24 mg/g). The TPC of grape seed was 20.02 mg/g, and suggested that it might be affected by varieties and extracting protocols [36]. The *β*-carotene bleaching method is based on the loss of yellow color of *β*-carotene because the free radical produced from linoleic acid is able to strongly destroy unsaturated *β*-carotene [37]. In this study, grape seed oil extracts were shown to be the most potent in the *β*-carotene bleaching assay compared to the other edible oils (Table 2). Among the identified phenolic acids, benzoic acid and vanillic acid were observed as the major phenolic compounds, and esculetin, isoquercetin, myricetin, luteolin, and kaempferol were the principal flavonoids in most edible oils. Benzoic acid is extensively used in preservatives, flavor enhancers, analgesics, antiseptics, and chemotherapeutics. Fisetin was found in sunflower (Table 5), while sinapic acid was detected only in cotton and perilla (Table 4). 

The antimicrobial activity of the edible oils was examined on two common bacteria—*S. aureus* and *E. coli*. The results demonstrated that all of the oils possessed antimicrobial activity on both *S. aureus* and *E. coli* (Table 6). Friedman et al. [38] reported that edible oils were widely accepted because they possessed unimaginable medicinal and pharmaceuticals properties, such as antimicrobial activity that could be employed against human pathogens. Rice bran possessed many bioactive compounds [39]. In this study, rice bran showed the highest activity on *S. aureus*. Moreover, the antibacterial activity of avocado oil extract was the strongest against *E. coli*. Neeman [40] reported that avocado possessed metabolites with antibacterial activity such as 1,2,4-trihydroxy-n-heptadeca-16-en that was extracted from avocado fruit and seeds. Other studies noted that avocado exerted strong antimicrobial activity, including the defensin PaDef which has a *γ*-core motif, which was also important for structure stabilization [41,42]. Furthermore, tannins, catechin flavones, and polyphenolics in seeds and immature fruits of avocado may also play a role in the antimicrobial activity [43]. 

The consumption of edible oils in Japan is increasing [23]; hence, the findings of this study provide useful information on the antioxidant and antimicrobial properties of the most available essential oils in Japanese markets. However, further analyses should be carried out by widening the sample collection and analyzing oils produced in controlled conditions (i.e., in laboratory) so as to eliminate the influence of different processing conditions, which might affect the results obtained in this study. Useful information on the antioxidant and antimicrobial properties, as well as chemical profiles, may help customers to have more choices in the consumption of the edible oils in Japan’s markets.

## Figures and Tables

**Table 1:** Fourteen edible oils and their origins.

Name	Part Used for Oils	Produced Companies
Sunflower	Seeds	Monte Bussan K.K., Tokyo, Japan
Safflower	Seeds	J-Oil Mills Inc., Tokyo, Japan
Canola	Seeds	The Nisshin Oillio Group, Ltd., Tokyo, Japan
Soybean	Seeds	Junko Inc., Tokyo, Japan
Cotton	Seeds	The Nisshin Oillio Group, Ltd., Tokyo, Japan
Grape	Seeds	The Nisshin Oillio Group, Ltd., Tokyo, Japan
Flax	Seeds	Nippon Flour Mills Co., Ltd., Tokyo, Japan
Perilla	Seeds	Ohta Oilmill Co., Ltd., Aichi, Japan
Avocado	Seeds	Benibana Food Co., Ltd., Tokyo, Japan
Chia	Seeds	K-Tac Planners Co., Ltd., Tokyo, Japan
Inca inchi	Seeds	Arcoiris Company, Chiba, Japan
Perillartine	Plants	Benibana Food Co., Ltd., Tokyo, Japan
Sesame	Seeds	Kadoya Sesame Mills Co., Ltd., Tokyo, Japan
Rice bran	Bran	Tsuno Food Industrial Co., Ltd., Wakayama, Japan

**Table 2:** 1,1-Diphenyl-2-picrylhydrazyl (DPPH) scavenging and *β*-carotene bleaching activities of the edible oils.

Name	DPPH Scavenging Activity (IC_50_ (mg/mL)	*β*-Carotene/Linoleic Acid Inhibition (LPI%)
Sunflower	0.2 ± 0.0 ^d^	82.4 ± 2.3 ^a,b,c,d^
Safflower	0.4 ± 0.0 ^c,d^	76.6 ± 4.9 ^b,c,d,e^
Canola	0.4 ± 0.1 ^c,d^	79.0 ± 1.3 ^a,b,c,d,e^
Soybean	0.362 ± 0.0 ^c,d^	72.5 ± 15.7 ^d,e^
Cotton	0.4 ± 0.027 ^c,d^	91.1 ± 1.2 ^a,b^
Grape	0.6 ± 0.0 ^c,d^	92.3 ± 3.1 ^a^
Flax	2.4 ± 0.2 ^b^	78.5 ± 0.0 ^a,b,c,d,e^
Perilla	0.8 ± 0.0 ^c^	78.9 ± 3.1 ^a,b,c,d,e^
Avocado	6.2 ± 0.4 ^a^	68.8 ± 3.0 ^e^
Chia	6.1 ± 0.4 ^a^	67.9 ± 0.7 ^e^
Inca inchi	0.4 ± 0.0 ^c,d^	88.1 ± 5.5 ^a,b,c^
Perillartine	2.80 0.1 ^b^	77.0 ± 5.2 ^a,b,c,d,e^
Sesame	0.5 ± 0.0 ^c,d^	72.9 ± 2.6 ^c,d,e^
Rice bran	0.5 ± 0.0.0 ^c,d^	85.0 ± 1.9 ^a,b,c,d^

Values represent means ± SE (standard errors) (*n* = 3). Different letters in similar column indicate significant difference (*p* < 0.05). LPI: lipid peroxidation inhibition.

**Table 3:** Total phenolic and flavonoid contents of 14 edible oil extracts.

Name	TPC (mg GAE/g OE)	TFC (mg RE/g OE)
Sunflower	4.39 ± 0.20 ^h,i^	0.06 ± 0.01 ^d,e^
Safflower	1.76 ± 0.29 ^j^	0.03 ± 0.01 ^e^
Canola	3.01 ± 0.14 ^i,j^	0.07 ± 0.01 ^c,d,e^
Soybean	3.23 ± 0.08 ^h,i,j^	0.03 ± 0.01 ^e^
Cotton	8.22 ± 0.39 ^g^	0.05 ± 0.01 ^d,e^
Grape	15.56 ± 0.24 ^d^	0.10 ± 0.02 ^c,d,e^
Flax	39.16 ± 1.03 ^a^	0.16 ± 0.05 ^b,c,d^
Perilla	18.07 ± 1.11 ^c^	0.15 ± 0.03 ^b,c,d^
Avocado	11.31 ± 0.37 ^f^	0.19 ± 0.02 ^b,c^
Chia	4.86 ± 0.30 ^h^	0.11 ± 0.03 ^c,d,e^
Inca inchi	13.29 ± 0.05 ^e^	0.34 ± 0.12 ^a^
Perillartine	20.38 ± 0.17 ^b^	0.12 ± 0.03 ^c,d,e^
Sesame	10.46 ± 0.50 ^f^	0.26 ± 0.01 ^a,b^
Rice bran	19.59 ± 1.25 ^b,c^	0.11 ± 0.01 ^c,d,e^

Values represent means ± SE (standard errors) (*n* = 3). Different letters in a column indicate significant difference (*p* < 0.05). GAE: gallic acid equivalent; OE: oil extract; RE: rutin equivalent; TPC: total phenolic content; TFC: total flavonoid content.

**Table 4:** Quantification of phenolic acids (mg/mL) detected in the 14 edible oils.

Samples	Compounds								
	ChA	*p*-HA	VA	FeA	SiA	*p*-CA	BA	EA	Total
Su-0	-	-	-	-	-	0.042 ± 0.027 ^a^	0.380 ± 0.085 ^a,b,c^	-	0.422
Sa-0	-	-	-	-	-	0.005 ± 0.001 ^b^	0.226 ± 0.044 ^b,c^	-	0.231
Ca-0	-	-	-	-	-	-	0.213 ± 0.050 ^b,c^	-	0.213
S0-B	-	0.007 ± 0.003 ^a^	-	0.004 ± 0.001 ^b^	-	0.005 ± 0.001 ^b^	-	0.009 ± 0.002 ^b^	0.025
Co-S	0.040 ± 0.013 ^a^	-	-	-	0.061 ± 0.072 ^a^	0.005 ± 0.001 ^b^	0.403 ± 0.303 ^a,b,c^	-	0.509
Gr-S	-	-	0.038 ± 0.055 ^a^	-	-	-	0.071 ± 0.018 ^c^	-	0.109
Fl-S	0.079 ± 0.023 ^a^	-	0.019 ± 0.001 ^a^	-	-	-	0.423 ± 0.083 ^a,b^	0.019 ± 0.001 ^a^	0.540
Pe-S	0.062 ± 0.027 ^a^	-	0.043 ± 0.029 ^a^	0.012 ± 0.004 ^a^	0.008 ± 0.002 ^a^	-	-	-	0.139
Av-0	-	0.017 ± 0.001 ^a^	0.031 ± 0.003 ^a^	-	-	-	0.607 ± 0.201 ^a^	0.007 ± 0.001 ^b^	0.662
Ch-S	-	0.007 ± 0.007 ^a^	0.023 ± 0.004 ^a^	-	-	-	0.351 ± 0.037 ^a,b,c^	-	0.381
IN-0	-	-	0.026 ± 0.001 ^a^	-	-	-	0.317 ± 0.016 ^a,b,c^	-	0.343
Pe-A	-	0.006 ± 0.004 ^a^	0.027 ± 0.001 ^a^	-	-	-	0.264 ± 0.013 ^b,c^	-	0.297
Se-0	-	-		0.008 ± 0.002 ^a,b^	-	-	0.152 ± 0.031 ^b,c^	-	0.160
Ri-0	-	-		0.009 ± 0.004 ^a,b^	-	-	0.147 ± 0.040 ^b,c^	-	0.156
	ns	ns	ns		ns	ns			

Values represent means ± SE (standard errors) (*n* = 3). Different letters in similar column indicate significant difference (*p* < 0.05). ChA: chlorogenic acid; *p*-HA: *p*-hydroxybenzoic acid; VA: vanillic acid; FeA: ferulic acid; SiA: sinapic acid; *p*-CA: *p*-coumaric acid; BA: benzoic acid; EA: ellagic acid. Su-0: sunflower oil; Sa-0: safflower oil; Ca-0: canola oil; S0-B: soybean oil; Co-S: cotton seed oil; Gr-S: grape seed oil; Fl-S: flax seed oil; Pe-S: perilla seed oil; Av-0: avocado oil; Ch-S: chia seed oil; IN-0: Inca inchi oil; Pe-A: perillartine oil; Se-0: sesame oil: Ri-0: rice bran oil; -: not detected; ns: not significant.

**Table 5:** Quantification of major flavonoid compounds (mg/mL) detected in the 14 edible oils.

Samples	Compounds							Total
	Es	Is	My	Fi	Lu	Ka	Rh	
Su-0	3.553 ± 0.426 ^a^	2.257 ± 0.803 ^a^	-	2.900 ± 0.100 ^a^	0.862 ± 0.760 ^b,c^	0.731 ± 0.271 ^a^	-	10.303
Sa-0	-	0.280 ± 0.033 ^b^	-	-	-	-	-	0.28
Ca-0	0.761 ± 0.047 ^c^	0.227 ± 0.033 ^b^	-	-	-	-	-	0.988
S0-B	-	-	6.133 ± 4.007 ^a,b^	-	0.941 ± 0.599 ^b,c^	-	-	7.074
Co-S	-	-	5.979 ± 2.925 ^a,b^	-	0.728 ± 0.420 ^b,c^	-	-	6.707
Gr-S	1.975 ± 0.074 ^b^	-	4.092 ± 0.144 ^b^	-	0.409 ± 0.022 ^c^	-	3.613 ± 0.180 ^a^	10.089
Fl-S	0.826 ± 0.104 ^c^	-	-	-	-	0.239 ± 0.016 ^c^	2.127 ± 0.015 ^b^	3.192
Pe-S	-	-	9.947 ± 1.236 ^a^	-	2.595 ± 0.199 ^a^	-	-	12.542
Av-0	-	-	4.517 ± 1.357 ^a,b^	-	1.607 ± 0.151 a^b,c^	-	-	6.124
Ch-S	-	-	3.986 ± 0.386 ^b^	-	1.718 ± 0.347 ^a,b^	-	-	5.704
IN-0	-	-	4.600 ± 0.702 ^a,b^	-	1.570 ± 0.532 ^a,b,c^	-	-	6.170
Pe-A	1.974 ± 0.508 ^b^	1.096 ± 0.429 ^b^	-	-	-	0.452 ± 0.112 ^a,b,c^	-	3.522
Se-0	0.950 ± 0.161 ^c^	0.255 ± 0.101 ^b^	-	-	-	0.325 ± 0.078 ^b,c^	-	1.530
Ri-0	0.853 ± 0.171 ^c^	-	-	-	-	0.677 ± 0.0114 ^a,b^	-	1.530
				ns				

Values represent means ± SE (standard errors) (*n* = 3). Different letters in similar column indicate significant difference (*p* < 0.05). Es: esculetin; Is: isoquercetin; My: myricetin; Fi: fisetin; Lu: luteolin; Ka: kaempferol; Rh: rhamnetin. Su-0: sunflower oil; Sa-0: safflower oil; Ca-0: canola oil; S0-B: soybean oil; Co-S: cotton seed oil; Gr-S: grape seed oil; Fl-S: flax seed oil; Pe-S: perilla seed oil; Av-0: avocado oil; Ch-S: chia seed oil; IN-0: Inca inchi oil; Pe-A: perillartine oil; Se-0: sesame oil: Ri-0: rice bran oil; ns: not significant; -: not detected.

**Table 6:** Antimicrobial activity of the 14 edible oils.

Sample	*S. aureus*			*E. coli*		
	0.10 mg/mL	0.05 mg/mL	0.01 mg/mL	0.10 mg/mL	0.05 mg/mL	0.01 mg/mL
Su-0	8.667 ± 2.517 ^b,c^	7.000 ± 1.732 ^b^	6.333 ± 0.577 ^b^	10.333 ± 1.528 ^c,d,e^	8.333 ± 2.309 ^b,c^	6.667 ± 0.577 ^b^
Sa-0	14.000 ± 5.000 ^a,b,c^	10.667 ± 5.033 ^b^	9.333 ± 3.512 ^b^	13.000 ± 3.000 ^b,c,d^	9.667 ± 2.082 ^b,c^	8.667 ± 2.082 ^b^
Ca-0	12.667 ± 2.517 ^a,b,c^	8.667 ± 1.155 ^b^	6.667 ± 0.577 ^b^	13.667 ± 0.577 ^b,c,d^	9.667 ± 0.577 ^b,c^	7.000 ± 0.001 ^b^
S0-B	13.000 ± 1.000 ^a,b,c^	12.333 ± 1.155 ^b^	7.333 ± 0.577 ^b^	11.333 ± 2.082 ^c,d^	11.667 ± 2.309 ^b^	7.000 ± 0.001 ^b^
Co-S	17.667 ± 1.155 ^a,b^	12.000 ± 1.000 ^b^	7.333 ± 1.155 ^b^	13.667 ± 1.528 ^b,c,d^	12.000 ± 1.000 ^b^	8.000 ± 1.000 ^b^
Gr-S	16.667 ± 6.807 ^a,b^	12.000 ± 4.583 ^b^	7.000 ± 1.000 ^b^	14.667 ± 3.786 ^b,c^	10.000 ± 2.000 ^b,c^	9.667 ± 2.082 ^b^
Fl-S	15.333 ± 1.155 ^a,b,c^	9.667 ± 2.082 ^b^	7.667 ± 0.577 ^b^	12.667 ± 1.155 ^b,c,d^	11.000 ± 1.000 ^b^	8.000 ± 1.000 ^b^
Pe-S	11.000 ± 1.000 ^a,b,c^	7.667 ± 1.528 ^b^	7.667 ± 0.577 ^b^	11.667 ± 1.528 ^c,d^	9.667 ± 1.528 ^b,c^	8.667 ± 2.517 ^b^
Av-0	14.667 ± 4.041 ^a,b,c^	9.000 ± 1.732 ^b^	6.667 ± 0.577 ^b^	17.000 ± 1.000 ^b^	10.667 ± 0.577 ^b,c^	7.667 ± 1.528 ^b^
Ch-S	16.000 ± 1.732 ^a,b^	13.000 ± 3.606 ^a,b^	9.333 ± 2.517 ^b^	11.000 ± 1.000 ^c,d,e^	8.667 ± 0.577 ^b,c^	6.667 ± 1.155 ^b^
IN-0	10.333 ± 0.577 ^b,c^	8.667 ± 2.082 ^b^	6.333 ± 0.577 ^b^	12.333 ± 1.528 ^b,c,d^	10.667 ± 0.577 ^b,c^	8.667 ± 1.528 ^b^
Pe-A	12.333 ± 0.577 ^a,b,c^	8.667 ± 1.528 ^b^	7.000 ± 1.000 ^b^	9.333 ± 1.528 ^d,e^	8.000 ± 2.000 ^b,c^	7.000 ± 1.732 ^b^
Se-0	16.000 ± 6.557 ^a,b^	9.667 ± 3.055 ^b^	8.000 ± 2.646 ^b^	12.667 ± 0.577 ^b,c,d^	11.000 ± 0.000 ^b^	8.667 ± 2.309 ^b^
Ri-0	20.333 ± 2.082 ^a^	11.667 ± 0.577 ^b^	8.667 ± 1.155 ^b^	15.333 ± 1.155 ^b,c^	9.667 ± 3.512 ^b,c^	7.333 ± 1.528 ^b^
P-C	20.000 ± 1.000 ^a^	20.000 ± 1.000 ^a^	20.000 ± 1.000 ^a^	23.000 ± 1.000 ^a^	23.000 ± 1.000 ^a^	23.000 ± 1.000 ^a^
N-C	6.000 ± 0.001 ^c^	6.000 ± 0.001 ^b^	6.000 ± 0.001 ^b^	6.000 ± 0.001 ^e^	6.000 ± 0.001 ^c^	6.000 ± 0.001 ^b^

Values represent means ± SE (*n* = 3). Different letters in the same column indicate significant difference (*p* < 0.05). P-C: positive control, N-C: negative control. Su-0: sunflower oil; Sa-0: safflower oil; Ca-0: canola oil; S0-B: soybean oil; Co-S: cotton seed oil; Gr-S: grape seed oil; Fl-S: flax seed oil; Pe-S: perilla seed oil; Av-0: avocado oil; Ch-S: chia seed oil; IN-0: Inca inchi oil; Pe-A: perillartine oil; Se-0: sesame oil: Ri-0: rice bran oil.

**Table 7:** Correlation coefficients among examined factors in the 14 edible oils.

Factors	TPC	TFC	DPPH	*β*-Carotene	IPA	IF	*S. aureus*	*E. coli*
TPC		0.1232	0.0118	0.0313	0.3235	0.0001	0.0431	0.0182
TFC			0.0224	0.0001	0.0314	0.0006	0.0159	0.0029
DPPH				0.401	0.3322	0.0001	0.0221	0.0007
*β*-Carotene					0.0113	0.0647	0.0108	0.0445
IPA						0.0001	0.0002	0.0003
IF							0.1496	0.0709

Mean in column is expressed by a linear value (*r*^2^) at *p* < 0.05. Abbreviations: TPC: total phenol content; TFC: total flavonoid content; DPPH: DPPH radical scavenging activity; *β*-carotene: *β*-carotene bleaching activity; IPA: total amount of individual phenolic acids; IF: total amount of individual flavonoids; *S. aureus* and *E. coli*: antimicrobial activity on *S. aureus* and *E. coli*.
